# Anti-cancer properties of boswellic acids: mechanism of action as anti-cancerous agent

**DOI:** 10.3389/fphar.2023.1187181

**Published:** 2023-08-03

**Authors:** Vijay Laxmi Trivedi, Ruchi Soni, Praveen Dhyani, Priyanka Sati, Silvia Tejada, Antoni Sureda, William N. Setzer, Ahmad Faizal Abdull Razis, Babagana Modu, Monica Butnariu, Javad Sharifi-Rad

**Affiliations:** ^1^ High Altitude Plant Physiology Research Centre (HAPPRC), HNB. Garhwal University (A Central University), Srinagar Garhwal, Uttarakhand, India; ^2^ Regional Centre for Organic and Natural Farming, Ghaziabad, Uttar Pradesh, India; ^3^ Institute for Integrated Natural Sciences, University of Koblenz, Koblenz, Germany; ^4^ Department of Biotechnology, Kumaun University, Bhimtal, Uttarakhand, India; ^5^ Laboratory of Neurophysiology, Department of Biology, University of the Balearic Islands, Palma de Mallorca, Spain; ^6^ Health Research Institute of Balearic Islands (IdISBa), Palma de Mallorca, Spain; ^7^ CIBER Fisiopatología de la Obesidad y Nutrición (CIBEROBN), Instituto de Salud Carlos III (ISCIII), Madrid, Spain; ^8^ Research Group in Community Nutrition and Oxidative Stress, University of the Balearic Islands—IUNICS, Palma de Mallorca, Spain; ^9^ Aromatic Plant Research Center, Lehi, UT, United States; ^10^ Department of Chemistry, University of Alabama in Huntsville, Huntsville, AL, United States; ^11^ Department of Food Science, Faculty of Food Science and Technology, Universiti Putra Malaysia, Selangor, Malaysia; ^12^ Natural Medicines and Products Research Laboratory, Institute of Bioscience, Universiti Putra Malaysia, Selangor, Malaysia; ^13^ Department of Biochemistry, Faculty of Science, University of Maiduguri, Maiduguri, Nigeria; ^14^ University of Life Sciences “King Mihai I” From Timisoara, Timis, Romania; ^15^ Facultad de Medicina, Universidad del Azuay, Cuenca, Ecuador

**Keywords:** *Boswellia*, cancer, bioactive compounds, apoptosis, triterpenes

## Abstract

With the advent of highly effective plant-based medications with few or no side effects, the use of phytomedicines against complex diseases such as cancer is becoming more widespread. The broadly recognized pentacyclic triterpenes known as boswellic acids (BAs) are derived from the oleogum resin, or frankincense, extracted from the plant species of the genus *Boswellia*. The frankincense mixture contains various BA types, each having a different potential and helping treat certain cancers. This review focuses on details regarding the traits of the BAs, their roles as anti-cancer agents, the mechanism underlying their activities, and the function of their semi-synthetic derivatives in managing and treating certain cancers. The review also explores the biological sources of BAs, how they are conserved, and how biotechnology might help preserve and improve *in vitro* BA production. The review concludes that the BAs and their semi-synthetic derivatives are effective against a broad spectrum of cancer cell lines. The detailed information in the review can be helpful for researchers to gain more information about BAs and BA-based medications for efficient and cost-effective cancer treatments.

## 1 Introduction

The discovery of several plant-based chemicals with anti-cancer potential reinstated the ancient traditional knowledge of herbal medicines with the support of scientific knowledge. The identification of compounds such as vinblastine, vincristine, and taxol as anti-cancerous agents laid the fundamentals for the discovery of new phytochemical anti-cancerous agents (Dhyani et al., 2022). In recent years, the finding of phytochemicals with potential anti-cancer activities with no or fewer side effects has been accelerated. One such class of compounds, known as boswellic acids, is extracted from the *Boswellia* genus and is extensively used to treat various other chronic diseases. These include haemolytic, spasmolytic, antiviral, anti-inflammatory, hepatoprotective, gastroprotective, and anti-microbial properties (Sun et al., 2006; Agrawal et al., 2011; Hussain et al., 2017). Boswellic acids (BAs) are pentacyclic triterpenes derived from the frankincense tree. *Boswellia serrata*, popularly referred to as white guggal, Indian olibanum, Salai Guggal, and dhup, is the main source of BAs (Havel et al., 2002; Qurishi et al., 2012), although *B. carteri* (Roy et al., 2019), *B. sacra*, and *B. papyrifera* are additional sources of BAs (Al-Harrasi et al., 2019). Usually, the gum resins of *Boswellia* species were employed for various purposes, such as adhesives, cosmetic preparations, coating materials, and incense used in cultural ceremonies and rituals. It is one of the most important and widely used ingredients in traditional Ayurvedic and Unani remedies, which are exceptionally successful in treating a variety of inflammatory, gastrointestinal, hormonal, and microbiological illnesses (Siddiqui, 2011). The BAs are separated from the gum resin frankincense, which is made up of essential oil, mucous, and a lipophilic portion. The grades and content of this resin vary according to the species of *Boswellia* used to extract it (Saraswati and Agrawal, 2012). The gum resin of *Boswellia* species contains up to 12 different types of BAs among which four major types of BAs are β-BA, A-β-BA, KBA, and AKBA with different pharmacological properties such as anti-cancer, anti-angiogenic, anti-tumour, apoptosis induction, anti-proliferative, and anti-inflammatory, among others (Liu et al., 2002). Nevertheless, not all BAs have an identical activity or potency (Liu et al., 2002; Yadav et al., 2012). For example, KBA and AKBA are the most effective at suppressing cytokine production and inhibiting the enzymes responsible for inflammatory reactions. As a result, these have been described as effective treatments for a variety of chronic conditions (Siddiqui, 2011; Roy et al., 2019).

BAs have been reported to be beneficial in both the prevention and treatment of various cancers such as breast, bladder, cervix, prostate, colorectal, head and neck, liver, lung, and pancreas (Roy et al., 2019). Several semi-synthetic derivatives of the different BAs, that show chemotherapeutic promise against diverse cancerous human cell lines, were also synthesized further to enhance the BAs’ anti-cancer action (Liu et al., 2002). Apoptosis, reducing angiogenesis of cancerous cells, obstructing blood flow to the tumour tissue, and down-regulating AKT phosphorylation are some of the mechanisms that BAs use to prevent cancer metastasis, depending on the type of cancer cells targeted (Liu et al., 2002; Uthaman et al., 2012).

This review examines BAs concerning their natural sources, conservation status, and *in vitro* biotechnological production potentials. This review also aims to describe the types of BAs, their chemical properties, semi-synthetic derivatives, and the mechanism of action of these compounds as anti-cancer agents. Scientific evidence supporting their categorization as anti-cancer substances is also presented, along with their modern and traditional applications as valuable drugs. The review will help increase the knowledge about plant-based anti-cancer therapeutics that includes various BAs and their derivatives.

## 2 General characterization of boswellic acid and its semi-synthetic derivatives

**FIGURE 1:**
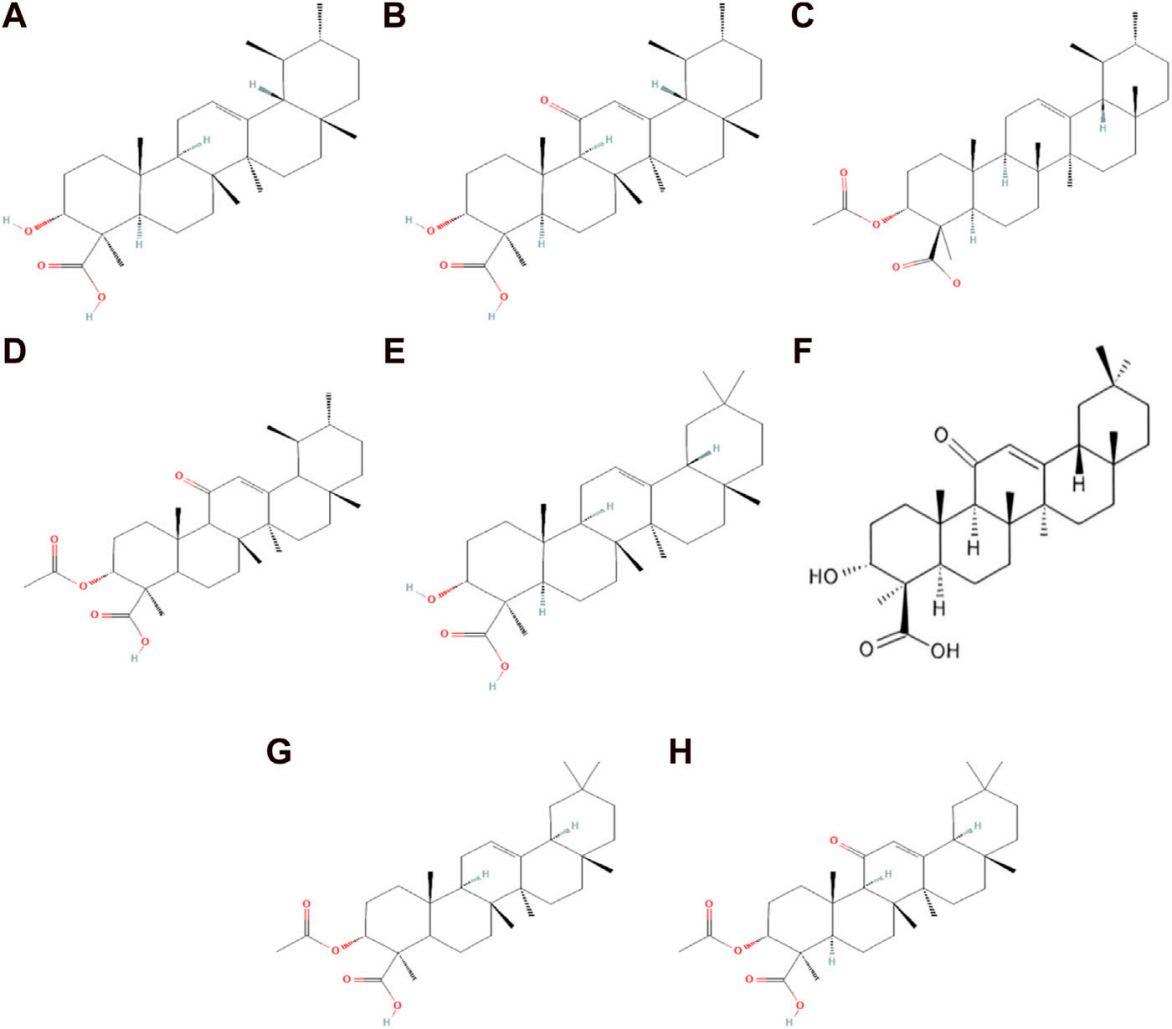
Chemical structure of ursane and oleanane-type boswellic acid. **(A)** β-Boswellic acid; **(B)**11-keto-β-boswellic acid; **(C)** acetyl-β-boswellic acid; **(D)** acetyl-11-keto-β-boswellic acid; **(E)** α-boswellic acid; **(F)** 11-keto-α-boswellic acid; **(G)** acetyl-α-boswellic acid; and **(H)** acetyl-11-keto-α-boswellic acid.

For centuries, frankincense (olibanum) extracted from the *Boswellia* tree, mainly from *B. serrata*, has been used as a source of BAs. Other species studied included *B. sacra, B. papyrifera*, and *B. carteri*, which are also used as BA sources worldwide (Al-Harrasi et al., 2019; Bongers et al., 2019). The resin of the *Boswellia* tree is composed of essential oil (5%–9%) and mucilage (6%–20%), the major component of the BAs, which has been quantified around 25%–35% of the resin acid mixture (Ennet, 2000; Al-Harrasi et al., 2021). BAs are a group of bioactive organic acids containing a pentacyclic triterpene and a β-carboxyl group at the C-4 position. BAs are grouped into two groups: the first one is ursane-type (β-BAs), and the second one is oleanane-type (α-BAs). Ursane-type BAs contain ursane triterpene skeleton-type and include β-boswellic acid (BA), 11-keto-β-boswellic acid (KBA), acetyl-β-boswellic acid (ABA), and acetyl-11-keto-β-boswellic acid (AKBA). Oleanane-type boswellic acid consisted oleanane structure and included α-boswellic acid (α-BA), 11-keto-α-boswellic acid (α-KBA), acetyl-α-boswellic acid (α-ABA), and acetyl-11-keto-α-boswellic acid (α-AKBA) (Shah et al., 2009; Al-Harrasi et al., 2019; Al-Harrasi et al., 2021; Schmiech et al., 2021) (Figure 1).

The resin containing the BAs is isolated from the *Boswellia* tree through wounding and subsequent tapping. These extraction procedures induced a chain of signaling process in the *Boswellia* tree involving gene expression and endogenous BAs production in the wounded location of the tree ultimately resulting in the clumpy frankincense (Khan et al., 2018). However, the BA content in the resin of the *Boswellia* genus varies at inter-species and intra-species levels. Studies showed considerable variation in BAs, their precursor content, and the type of BAs present in the resin of *B. serrata*, *B. sacra*, and *B. papyrifera* (Paul, 2012). For example, in the study of Paul (2012), *B. papyrifera* showed higher β-AKB concentration but lower concentrations of the other BAs and other secondary metabolites, whereas *B. serrata* showed lower β-KBA and β-AKBA concentrations and higher concentrations of α-BA and β-BA. Within a species, the BA content is greatly influenced by the micro- and macroclimatic conditions to which the *Boswellia* trees are subjected (Park et al., 2011). Geographical variations were also observed in various *Boswellia* spp. populations in the BA content of the resin (Al-Harrasi et al., 2018). Even the different tissues of the same tree showed variations in the BA content and the compositions in the studies; for instance, *B. sacra* roots were devoid of the BAs (Paul, 2012), whereas the leaves had trace amounts of β-ABA and β-AKBA. The amyrins are the BAs synthesized by the terpenoid biosynthetic pathway (MVA Pathway), the immediate precursor of the boswellic acids. β-Amyrin is the α-boswellic acid precursor, which is an oleanane, and α-amyrin is the β-boswellic acid precursor, which is an ursane. The different BA compounds extracted from the frankincense (resin) of *Boswellia* are listed in Table 1.

**TABLE 1:** Boswellic acids naturally found in *Boswellia* species.

Compound name	Chemical formula of the compound	Molecular weight of the compound (g/mol)
β-Boswellic acid	C_30_H_48_O_3_	456.7
11-Keto-β-boswellic acid	C_30_H_46_O_4_	470.7
3-Acetyl-β-boswellic acid	C_32_H_50_O_4_	498.7
11-Hydroxy-β-boswellic acid	C_30_H_48_O_4_	472.7
3-Acetyl-11-keto-β-boswellic acid	C_32_H_48_O_5_	512.7
3-*O*-Acetyl-11-hydroxy-β-boswellic acid	C_32_H_50_O_5_	514.7
3-*O*-Acetoxy-11-methoxy-β-boswellic acid	C_33_H_52_O_5_	528.7
9,11-Dehydro-β-boswellic acid	C_30_H_46_O_3_	454.7
3-*O*-Acetyl-9,11-dehydro-β-boswellic acid	C_32_H_48_O_4_	496.7
α-Boswellic acid	C_30_H_48_O_3_	456.7
11-Keto-α-Boswellic Acid	C_30_H_46_O_4_	470.7
3-*O*-Acetyl-9,11-dehydro-α-boswellic acid	C_32_H_48_O_4_	496.7
11-Hydroxy-α-boswellic acid	C_30_H_48_O_4_	472.7
9,11-Dehydro-α-boswellic acid	C_30_H_46_O_3_	454.7
3-*O*-Acetyl-α-boswellic acid	C_32_H_50_O_4_	498.7

The main problem with most of the BAs is their low bioavailability, particularly for AKBA and KBA, which, in turn, raises questions about the pharmacological relevance of their bioactivities in animal and human research (Du et al., 2015). Synthesis of new derivatives by chemical modification and biotransformation of BAs can be an option (Table 2). BA derivatives have been synthesized for the discovery of new potent drugs, particularly the anti-cancer and tumour suppressors (Meng et al., 2005; Chaturvedi et al., 2015; Koeberle et al., 2018; Serbian et al., 2018; Shamraiz et al., 2020).

**TABLE 2:** Derivatives of the boswellic acids with their pharmacological action.

Precursor BA	Derivative	Pharmacological actions	Type of study and model	References
KBA	3-*O*-naproxen-β-boswellic acid	Anti-inflammatory potential and anti-arthritic properties	*In vivo* study, carrageenan-induced mice model, carrageenan-induced rat model	Chaturvedi et al. (2015)
KBA	3-*O*-naproxen-11-keto-β-boswellic acid	Anti-inflammatory and anti-arthritic activities	*In vivo* study, carrageenan-induced mice model, carrageenan-induced rat model	Chaturvedi et al. (2015)
KBA	3-*O*-ibuprofen-β-boswellic acid	Anti-inflammatory activity	*In vivo* study, carrageenan-induced mice model, carrageenan-induced rat model	Chaturvedi et al. (2015)
KBA	3-*O*-aspirin-β-boswellic acid	Anti-inflammatory activity	*In vivo* study, carrageenan-induced mice model, carrageenan-induced rat model	Chaturvedi et al. (2015)
KBA	3-*O*-aspirin-11-keto-β-boswellic acid	Anti-inflammatory activity	*In vivo* study, carrageenan-induced mice model, carrageenan-induced rat model	Chaturvedi et al. (2015)
KBA	3-*O*-cinnamyl-11-keto-β-boswellic acid	Anti-inflammatory activity	*In vivo* study, carrageenan-induced mice model, carrageenan-induced rat model	Chaturvedi et al. (2015)
β-BA	11α-Hydroxy-β-boswellic acid	Inhibited 5-lipoxygenase and cathepsin G and promoted apoptosis	*In vitro*, intact human neutrophils and purified cathepsin G	Koeberle et al. (2018)
β-BA	11β-Hydroxy-β-boswellic acid	Weak 5-LO inhibitors	*In vitro*, intact human neutrophils	Koeberle et al. (2018)
β-BA	3*-O-*oxaloyl-β-boswellic acid	Inhibits cathepsin G and promotes apoptosis	*In vitro*, purified cathepsin G	Koeberle et al. (2018)
β-BA	3-*O*-succinoyl-β-boswellic acid	Weak 5-LO inhibitors	*In vitro*, intact human neutrophils	Koeberle et al. (2018)
KBA	3*-O-*succinoyl-11-keto-β-boswellic acid	Weak 5-LO inhibitors	*In vitro*, intact human neutrophils	Koeberle et al. (2018)
β-BA	3-*O*-glutaroyl-β-boswellic acid	Inhibits cathepsin G and promotes apoptosis	*In vitro*, purified cathepsin G	Koeberle et al. (2018)
KBA	3-*O*-glutaroyl-11-keto-β-boswellic acid	Weak 5-LO inhibitors	*In vitro*, intact human neutrophils	Koeberle et al. (2018)
β-BA	3-*O*-carboxymethyl-β-boswellic acid	Inhibits cathepsin G and promotes apoptosis	*In vitro*, purified cathepsin G	Koeberle et al. (2018)
KBA	3-*O*-carboxymethyl-11-keto-β-boswellic acid	Weak 5-LO inhibitors	*In vitro*, intact human neutrophils	Koeberle et al. (2018)
AKBA	2,3-Dehydro-11-keto-β-boswellic acid	Cytotoxic	*In vitro*, human tumour cell lines	Serbian et al. (2018)
AKBA	2α-Hydroxy-11-keto-β-boswellic acid	Cytotoxic	*In vitro*, mouse monocyte–macrophage RAW 264.7 cells	Wang et al. (2013)
AKBA	1α-Hydroxy-2,3-dehydro-11-keto-β-boswellic acid	Cytotoxic	*In vitro*, mouse monocyte–macrophage RAW 264.7 cells	Wang et al. (2013)
AKBA	11-Keto-β-boswellic acid methyl ester	Cytotoxic	*In vitro*, mouse monocyte–macrophage RAW 264.7 cells	Wang et al. (2013)
AKBA	2,3-Dehydro-11-keto-β-boswellic acid methyl ester	Cytotoxic	*In vitro*, mouse monocyte–macrophage RAW 264.7 cells	Wang et al. (2013)
α BA	2α-Cyano-3-en-X-one of methyl boswellates	Growth inhibition of the cancerous cells, cytotoxic, anti-inflammatory, and pro-differentiating activities	*In vitro*, cancer cell lines	Ravanan et al. (2011)
βBA	2β-Cyano-3-en-X-one of the methyl boswellates	Growth inhibition of the cancerous cells, cytotoxic, anti-inflammatory, and pro-differentiating activities	*In vitro*, mouse monocyte–macrophage RAW 264.7 cells	Wang et al. (2013)
βBA	3α-Propionyloxy-β-boswellic acid	Cytotoxicity toward human cancerous cell lines by suppressing the PI3K pathway	*In vitro*, human cancer cell lines and *in vivo*, murine tumour models (Swiss albino mice)	Qurishi et al. (2012)
βBA	3α-Butyryloxy-β-boswellic acid	PI3K-mediated apoptosis	*In vitro*, mouse monocyte–macrophage RAW 264.7 cells	Wang et al. (2013)
KBA	3-Cinnamoyl-11-keto-β-boswellic acid	Proapoptotic effects and anti-proliferative	*In vitro*, cancer cell lines, and *in vivo*, PC-3 prostate cancer xenografts	Morad et al. (2013)
KBA	7β-Hydroxy-11-keto-β-boswellic acid	NO production inhibition by LPS-induced mechanism without declining cell viability in macrophages (RAW 264.7)	*In vitro*, mouse monocyte–macrophage RAW 264.7 cells	Wang et al. (2013)
KBA	7β,22β-Dihydroxy-11-keto-β-boswellic acid	NO production inhibition by LPS-induced mechanism without declining cell viability in macrophages (RAW 264.7)	*In vitro*, mouse monocyte–macrophage RAW 264.7 cells	Wang et al. (2013)
Acetyl-α-boswellic acid	3α-Acetyl-11-keto-α-boswellic acid	Activates apoptosis in androgen-independent chemo-resistant cancerous cells *in vivo* and *in vitro* by caspase-3 activation and DNA fragmentation induction	*In vitro*, human PC-3 prostate cancer cells and *in vivo*, xenograft model	Büchele et al. (2006)
3-Acetyl-11-keto-β-boswellic acid	7β-Hydroxyl-3-acety-11-keto-β-boswellic acid	NO production inhibition by LPS-induced mechanism without declining cell viability in macrophages (RAW 264.7)	*In vitro*, RAW 264.7 macrophage cells	Sun et al. (2013)
3-Acetyl-11-keto-β-boswellic acid	7β,16α-Dihydroxy-3-acety-11-keto-β-boswellic acid	NO production inhibition by LPS-induced mechanism without declining cell viability in macrophages (RAW 264.7)	*In vitro*, RAW 264.7 macrophage cells	Sun et al. (2013)

## 3 Mechanism of anti-tumour action of boswellic acid

BAs have been investigated for decades and are found to exhibit robust anti-cancer properties *in vitro* and *in vivo* (Table 3). Different isomers and extracts from the acids exhibit anti-cancer characteristics with distinct mechanisms in many types of cancer. The mechanisms of activity of BAs comprise a variety of targets, including the enzymes of angiogenesis and others such as topoisomerases, 5-lipoxygenase (5-LO), cytochrome P450, and mitogen-activated protein kinase (MAPK, especially p38) which are either promoted or inhibited by BAs (Iram et al., 2017).

**TABLE 3:** Scientific studies emphasizing boswellic acids as a promising anti-cancer agent.

Boswellic acids	Type of cancer	Inhibitory activities on cell lines	Techniques to analyse the inhibitory activities	References
BAA (o- and b-boswellic acid acetate)	Metastatic, melanoma, fibrosarcoma	Mouse melanoma cells BI6F10 and human fibrosarcoma cell line HT-1080	MTT proliferation assay, cell viability analysis, gelatin zymography, topoisomerase-II catalytic assay, flow cytometry, and DNA fragmentation	Zhao et al. (2003)
AKBA	Prostate cancer	Inhibited cellular proliferation on LNCaP cell lines associated with reduced androgen receptor expression	Flow cytometry, MTT assay, transient transfection assay, electrophoretic mobility shift assay, and western blot analysis	Yuan et al. (2008)
AKBA	Colon cancer	Initiated apoptosis in HT 29 colon cancer cells	Flow cytometry and caspase assay on cell lines	Liu and Duan (2009)
AKBA	Pancreatic cancer	Induced cellular proliferation inhibition of AsPC-1, BxPC-3, and MIAPaCa-2	MTT proliferation assay, western blot, and immune histochemical analysis PANC-28, an orthotopic mouse model	Park et al. (2011)
BA	Breast cancer	Inhibited MCF-7 cell proliferation and potentiated the cell death	Caspase activity assay, cytokine ELISA assay, superoxide dismutase activity, glutathione essay, and catalase activity	Saraswati and Agrawal (2012)
BA	Colorectal cancer	Orthotopic mouse model	Proliferative index and nuclear factor-κB suppression	Yadav et al. (2012)
BSE (*Boswellia serrata* extract)	Pancreatic cancer	Cytotoxic effect on A375, MIA-PaCa, mouse melanoma, and human pancreatic cancer cell line	DNA fragmentation assay	Uthaman et al. (2012)
BA	Brain tumour	Exhibited antioxidant and analysing anti-inflammatory effects on p65/NF-κB and leukotriene B4 expression	Growth factors and interleukins production (vascular endothelial growth factor, IL-8 and IL-6, MMP-9, and CXCL-12)	Barbarisi et al. (2019)
AKBA	Lung cancer	Inhibited cell growth in A549, H460, H1299, and BEAS-2B cell lines	CCK-8 assay and flow cytometry	Lv et al. (2020)
11-Keto-boswellic acids (KBA)	Breast cancer	Three triple-negative breast cancer (TNBC) cell lines resistant to therapy were used to induce apoptosis in MDA-MB-231 xenografts *in vivo*	High-resolution mass spectrometry (HR-MS) and NMR spectroscopy and HPLC-MS/MS method developed	Schmiech et al. (2021)
KBA and AKBA	Lung cancer	Inhibited cell proliferation in eight cell lines	HPLC analysis, MTT assay, DPPH scavenging, and ABTS assays	Gupta et al. (2022)
β-Boswellic acid	Breast precancerous lesions	MCF-10A and MDA-MB-231 cell lines	Kyoto Encyclopedia of Genes and Genomes (KEGG) analysis, metabolism-related assays, and molecular docking analysis	Bie et al. (2022)

*B. serrata* gum resin extract (methanolic) showed the occurrence of triterpenoids, β-boswellic acid, and its analogues. Huang et al., 2000 reported that b-BA naturally occurring triterpenoids with their derivatives had been part of traditional medicine for cancer treatment (Huang et al., 2000). Several scientific studies have also shown *Boswellia*’s pentacyclic triterpenes as one of the most promising anti-cancer agents (Poeckel and Werz, 2006; Yuan et al., 2013; Al-Bahlani et al., 2020). AKBA and KBA are assessed by active inhibition of topoisomerase I and IIa, which restricts the growth of the cells and their proliferation and induces apoptosis through a pathway dependent on caspase-8 in human leukaemia, hepatoma, colon, and in a wide range of cancer cell lines (Xia et al., 2005; Suhail et al., 2011). Moreover, a chemoproteomic study based on mass spectrometry indicated that b-BAs also interact with the ribosomal proteins, inhibit protein synthesis, and thus further modulate cancer progression (Casapullo et al., 2016). Morphological alterations were noticed in treated HL-60 cells with AKBA, which is a signal of apoptosis of the cells. BA, 3-*O*-acetyl-β-boswellic acid, AKBA, and 3-*O*-acetyl-11-keto-boswellic acid showed anti-tumour activity and inhibition of DNA, RNA, and proteins synthesis in human leukaemia HL60 cells in a dose-dependent manner (Shao et al., 1998; Hoernlein et al., 1999).

AKBA showed cytotoxic action against three treatment-resistance triple-negative breast cancer cell lines (TNBC) and apoptosis in MDA-MB-231 xenografts in the *in vitro* study (Schmiech et al., 2021). AKBA diminished the viability of the cell in H460, H1299, A549, and BEAS-2B cell lines. In A549, cells caused cell cycle arrest at the G0/G1 phase, thus suppressing the clone formation and promoting the cellular apoptosis. It also reduced the expression of LC3A/B-I and LC3A/B-II, along with Beclin-1 proteins and inhibition of the signalling pathway of PI3K/Akt. It also suppressed protein expression and autolysosome formation (Lv et al., 2020). The latest β-isomer synthesized and characterized as 11-keto-boswellic acid (KBA) was discovered to have cytotoxic effects against three treatment-resistant triple-negative breast cancer (TNBC) cell lines *in vitro* and to cause apoptosis in MDA-MB-231 xenografts *in vivo* (Schmiech et al., 2021).

Recently, β-BA has been shown to inhibit precancerous breast lesions by suppressing the glycolysis pathway and reducing ATP production in MCF-10AT cells without damaging normal MCF-10A. It is also observed to suppress glycolysis which activates the AMPK pathway and inhibits the mTOR pathway to limit MCF-10AT proliferation. In the same study, analysis using molecular docking suggested that the target of β-BA might be GLUT1. The GLUT1 forced expression could rescue the suppression of glycolysis and induce survival checks on MCF-10AT (Bie et al., 2022).

**FIGURE 2:**
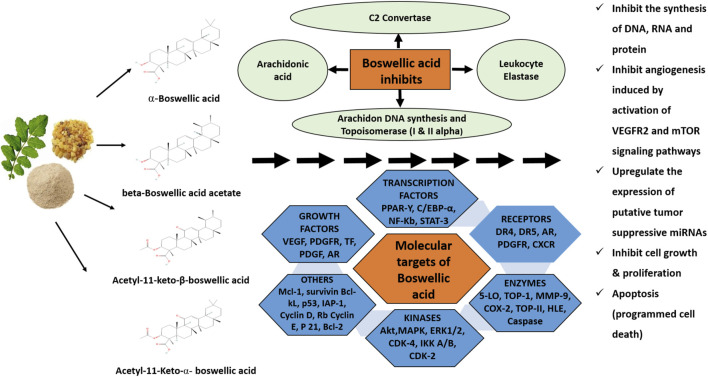
Schematic diagram of mechanisms of the boswellic acids extracted from the plant *Boswellia serrata*. These acids exhibit anti-cancer activities against different cancers by acting on numerous targets like 5-lipoxygenase (5-LO), topoisomerases, angiogenesis, and cytochrome p450 enzymes which finally lead to a reduction in cancer cell growth and apoptosis.

In another study, the effect of β-BA and AKBA has examined in nine human glioma stem-like cells and five glioma-initiating cell lines to analyse the acute growth inhibitory mechanism. The same study includes the anti-clonogenic characteristics along with the application of temozolomide (TMZ) or irradiation. The findings were correlated with previous findings indicating BA cytotoxicity in glioblastoma at low molecular concentrations. A significant synergistic action after application with irradiation and transcranial magnetic stimulation (TMS) was also observed (Schneider and Weller, 2016). These studies have provided insights into the different underlying mechanisms acquired by BAs for their anti-tumour actions. These findings can support the future development of their prospective as anti-inflammatory and anti-cancer drugs. The detailed mechanism of action of BAs as promising anti-cancer agents is depicted in the schematic diagram shown in Figure 2.

## 4 Studies that confirmed the anti-cancer properties of boswellic acids

BAs extracted from the plant *Boswellia serrata* are considered as the essential active constituents to treat many inflammatory diseases, either acute or chronic. Along with their potential as anti-arthritic, anti-asthmatic, anti-rheumatic, anti-diarrhoeal, and anti-hyperlipidemic, actions they also possess anti-microbial, hepatoprotective, analgesic, immunomodulatory actions, and anti-cancer characteristics. These acids have been found to exhibit very effective anti-inflammatory as well as anti-cancer activities in different models both *in vitro* and *in vivo*. They showed anti-cancer potential against a range of malignant tumours, and many semi-synthetic BAs illustrate outstanding cytotoxic effects (Hussain et al., 2021).

### 4.1 Cytotoxic effect of boswellic acid on colorectal cancer

Colorectal cancer is a multifaceted disease with epigenetic and genetic mutations in a wide range of oncogenes and tumour suppressor genes (Parmar and Easwaran, 2022). AKBA shows chemo-preventive characteristics capable of targeting principle oncogenic proteins, including 5-lipoxygenase and nuclear factor-kappa B. These BAs are known to modulate specific microRNA (miRNA) pathways due to their chemo-preventive effects. In the pathways, let-7 and miR-200 are both putative tumour-suppressive miRNAs. AKBA showed significantly upregulated expression of both families in various colorectal cancer cell lines. miRNA knockdown has been shown to inhibit let-7 and enable increased cancer cell propagation, migration, and invasion. AKBA modulates the expression of various downstream targets of the miR-200 and let-7 families (vimentin, CDK6, and E-cadherin). Similar findings of inducing modulation of these downstream genes have been observed in CRC cells orthotopically implanted in nude mice. This study gives novel evidence for the ability of BAs to regulate cellular epigenetic mechanisms that emphasise their anti-cancer characteristics and further highlight their potential in the prevention and treatment of CRC (Takahashi et al., 2012).

### 4.2 Anti-tumor effect of boswellic acid in human colonic adenocarcinoma

Ranjbarnejad et al. (2017) investigated the methanolic extract of Boswellia serrata for its anti-cancer activity on human colon cancer cells. This study establishes that the methanolic extract decreased the expression of cyclooxygenase-2 gene and its terminal end products, such as microsomal prostaglandin E synthase-1 (mPGES-1), vascular endothelial growth factor (VEGF), C-X-C chemokine receptor type 4 (CXCR4), matrix metalloproteinase-2 (MMP-2), MMP-9, and hypoxia-inducible factor-1 (HIF-1). The study, therefore, suggested that the *B. serrata* extract can be a potential agent to inhibit the proliferation, angiogenesis, and migration in colorectal cancer. Similarly, another study (Wang et al., 2018) also finds that BA can be used for the growth suppression of HCT-116 colon cancer cells. With an IC50 value of 15 uM, BA altered the Bax/Bcl-2 ratio in the HCT-116 cells. Therefore, a general understanding can be developed of the usage of BA as an anti-cancerous agent for human colon cancer, provided further *in vivo* detailed studies are performed.

### 4.3 Role of boswellic acid in growth suppression of human pancreatic tumours and its metastasis

BA acts as a growth suppressor for human pancreatic tumours in a mouse model by interacting with multiple targets and also limiting its metastasis. AKBA activity was studied in an *in vitro* model of orthotopic nude mice against human PaCa; it revealed that AKBA inhibited the proliferation of four PaCa cell lines. It comprised PANC-28, AsPC-1, and with p53 and K-Ras mutations, BxPC-3 with wild-type K-Ras and p53 mutation were also included. AKBA also inhibited the metastasis of the PaCa in the liver, spleen, and lungs in the same mouse model. The study indicated the potential of AKBA as an anti-tumour agent that exhibited an ability to suppress human pancreatic tumour growth and metastasis with multiple target modulations (Park et al., 2011).

### 4.4 Inhibitory activity of boswellic acids against human leukaemia

Four BAs of *B. serrata*: β-boswellic acid, 11-keto-β-boswellic acid, 3-O-acetyl-β-boswellic acid, and 3-O-acetyl-11-keto-β-boswellic acid were evaluated for their anti-cancer properties. They were examined to restrict the DNA, RNA, and protein synthesis in human leukaemia HL-60 cells. 3-*O*-Acetyl-11-keto-β-boswellic acid significantly inhibited the synthesis of DNA, RNA, and proteins. This compound had an irreversible effect on DNA synthesis, inhibiting the HL-60 cellular growth without affecting cell viability. The result of the study revealed a significant potential of this compound in the regulation of human leukaemia proliferation (Shao et al., 1998).

### 4.5 Apoptotic effect of boswellic acid in liver cancer cells

Only one study explored the anti-proliferative and apoptotic effect on Hep G2 liver cancer cells of keto-β-boswellic acid and acetyl-keto-β-boswellic acid. Following their application on the cell’s DNA synthesis, apoptosis and cell proliferation were examined. The apoptotic pathway was explored employing specific caspase inhibitors, which revealed the decreased cell viability and thymidine amalgamation and enlarged percentage of sub-G1 in the G1 phase. BAs significantly influenced apoptosis, complemented by the activation of these caspase inhibitors. Hence, the study led to the possibility of using the BAs for anti-cancer and anti-proliferation effects in the liver Hep G2 cells (Liu et al., 2002).

### 4.6 Apoptosis in prostate cancer cells *in vitro* and *in vivo* by boswellic acid

In androgen-independent PC-3 cells, a chemo-resistant prostate cancerous line, acetyl-β-boswellic acid, and acetyl-11-keto-β-boswellic acid inhibited their growth. They promoted the death of the cell *in vitro* as well as *in vivo* models (Syrovets et al., 2005). For analysing apoptosis, parameters like DNA fragmentation and mitochondrial cytochrome C release were examined in cultured PC-3 cells. The underlying molecular mechanism involved the inhibition of signalling of the NF-κB (constitutively activated) by IκB kinase (IKK) activity interruption. The IKK inhibition showed specificity because the signalling through the interferon-stimulated response element remained unchanged. The study was further confirmed in nude mice carrying PC-3 tumours, where the systemic application of AKβBA-γ- cyclodextrin reduced tumour growth. This treatment also activated apoptosis without detectable systemic toxicity. AKβBA and related compounds acting on IKK provide a novel approach for treating chemo-resistant human tumours, including androgen-independent human prostate cancers.

## 5 Conclusion

BAs and their semi-synthetic derivatives are effective against a broad spectrum of cancer cell lines. They have a minimal potential for resistance due to the multiple ways they operate in the cancer cell lines. The ability of BAs to control cellular epigenetic mechanisms highlights their anti-cancer properties, as they promote apoptosis in cancer cells and inhibit the malignant primary metabolic pathways and DNA, RNA, and protein synthesis. BAs are only present in the *Boswellia* genus, but they display several kinds and contents according to the species. Globally, the survival of the natural sources of frankincense is threatened by over-extraction to obtain BAs and other anthropogenic factors, including climate change. Seed dormancy and slower growth rate make it worse, so more *in vitro* conservation methods are required to protect these plant species. More research is necessary to develop technology for *in vitro* production of BAs from *Boswellia* spp., as well as more clinical trials and scientific studies to validate its anti-cancer potential and obtain novel cancer treatment.

## Abbreviations

BA, boswellic acid; BBA, β-boswellic acid; KBA, 11-keto-β-boswellic acid; ABA, acetyl-β-boswellic acid; AKBA, acetyl-11-keto-β-boswellic acid; AMPK, AMP-activated protein kinase; GLUT1, glucose transporter 1 protein; mTOR, mammalian target of rapamycin; TMS, transcranial magnetic stimulation; EGFR, epidermal growth factor receptor; PVP, polyvinylpyrrolidone.
